# Impacts of Filtration on Contrast-Detail Detectability of an X-ray Imaging System

**DOI:** 10.1155/IJBI/2006/95754

**Published:** 2006-04-03

**Authors:** Qirong Zhang, John Rong, Xizeng Wu, Yuhua Li, Wei R. Chen, Hong Liu

**Affiliations:** ^1^ Center for Bioengineering and School of Electrical and Computer Engineering, University of Oklahoma, Norman, OK 73019, USA; ^2^ Department of Radiological Sciences, University of Oklahoma Health Science Center, Oklahoma City, OK 73104, USA; ^3^ Department of Radiology, University of Alabama at Birmingham, Birmingham, AL 35294, USA; ^4^ Department of Physics and Engineering, University of Central Oklahoma, Edmond, OK 73034, USA

## Abstract

The purpose of this study is to investigate the impacts of added filtration on the contrast-detail detectability of a digital X-ray imaging system for small animal studies. A digital X-ray imaging system specifically designed for small animal studies was used. This system is equipped with a micro X-ray source with a tungsten target and a beryllium window filtration and a CCD-based digital detector. Molybdenum filters of 0 mm, 0.02 mm, and 0.05 mm in thickness were added. The corresponding X-ray spectra and contrast-detail detectabilities were measured using two phantoms of different thicknesses simulating breast tissue under different exposures. The added Mo filters reduced the low-energy as well as the high-energy photons, hence providing a narrowband for imaging quality improvement. In the experiments with a 1.15 cm phantom, the optimal image detectability was observed using 22 kVp and the 0.05 mm Mo filter. With the 2.15 cm phantom, the best detectability was obtained with 22 kVp and the 0.02 mm Mo filter. Our experiments showed that appropriate filtrations could reduce certain low- and high-energy components of X-ray spectra which have limited contributions to image contrast. At the same time, such filtration could improve the contrast-detail detectability, particularly at relatively low kVp and high filtration. Therefore, optimal image quality can be obtained with the same absorbed radiation dose by the subjects when appropriate filtration is used.


Hindawi Publishing Corporation
International Journal of Biomedical Imaging
Volume 2006, Article ID 95754, Pages 1–8
DOI 10.1155/IJBI/2006/95754

### Impacts of Filtration on Contrast-Detail Detectability of an X-ray Imaging System

**Qirong Zhang,[1] John Rong,[2] Xizeng Wu,[3] Yuhua Li,[1] Wei R. Chen,[4] and Hong Liu[1]**

[1] *Center for Bioengineering and School of Electrical and Computer Engineering, University of Oklahoma, Norman, OK 73019, USA*
[2] *Department of Radiological Sciences, University of Oklahoma Health Science Center, Oklahoma City, OK 73104, USA*
[3] *Department of Radiology, University of Alabama at Birmingham, Birmingham, AL 35294, USA*
[4] *Department of Physics and Engineering, University of Central Oklahoma, Edmond, OK 73034, USA*

Received 29 August 2005; Revised 8 November 2005; Accepted 2 December 2005

Recommended for Publication by Seung Wook Lee

The purpose of this study is to investigate the impacts of added filtration on the contrast-detail detectability of a digital X-ray imaging system for small animal studies. A digital X-ray imaging system specifically designed for small animal studies was used. This system is equipped with a micro X-ray source with a tungsten target and a beryllium window filtration and a CCD-based digital detector. Molybdenum filters of 0 mm, 0.02 mm, and 0.05 mm in thickness were added. The corresponding X-ray spectra and contrast-detail detectabilities were measured using two phantoms of different thicknesses simulating breast tissue under different exposures. The added Mo filters reduced the low-energy as well as the high-energy photons, hence providing a narrowband for imaging quality improvement. In the experiments with a 1.15 cm phantom, the optimal image detectability was observed using 22 kVp and the 0.05 mm Mo filter. With the 2.15 cm phantom, the best detectability was obtained with 22 kVp and the 0.02 mm Mo filter. Our experiments showed that appropriate filtrations could reduce certain low- and high-energy components of X-ray spectra which have limited contributions to image contrast. At the same time, such filtration could improve the contrast-detail detectability, particularly at relatively low kVp and high filtration. Therefore, optimal image quality can be obtained with the same absorbed radiation dose by the subjects when appropriate filtration is used.

Copyright © 2006 Qirong Zhang et al. This is an open access article distributed under the Creative Commons Attribution License, which permits unrestricted use, distribution, and reproduction in any medium, provided the original work is properly cited.

### 1. INTRODUCTION

When X-ray images are acquired, both patient radiation dose and image quality should be considered. Low-energy X-ray photons of an entrance spectrum penetrate tissue poorly and may not reach the image detector for useful diagnostic information. These attenuated X-ray photons only contribute to patient's radiation dose and should be selectively removed from the X-ray beam by adding appropriate filtration between the X-ray source and the patients. Contributing less to the patient's radiation dose, high-energy X-ray photons may at the same time result in poor image contrast. Appropriate filtration can remove not only low but also high-energy photons in the X-ray beam. On the other hand, if the total filtration is not selected properly, overfiltration mainly reduces the number of photons that reach the image detector. Lower beam intensity will degrade the image quality as reflected by lower signal-to-noise ratio (SNR). Therefore, the material and thickness of filters need to be carefully selected to satisfy both patient radiation dose and image quality considerations so that good performance of an X-ray imaging system can be achieved [1, 2].

The shape of X-ray spectra reflects the energy distribution of X-ray photons [3–5]. It determines the relevant radiation output characteristics and is important in X-ray imaging. Beaman et al. investigated the optimal X-ray spectra for mammography [3, 5]. They analyzed the influence of kVp setting, filter material, and filter thickness on the shape of X-ray spectra. The proper choice of the shape of the X-ray spectrum incident upon the breast can yield an improved image SNR for a given radiation dose [4]. The mean energy of the X-ray spectrum is not a good guide as to whether the spectrum is the appropriate one. If the spectrum is too broad, the low-energy X-rays contribute little to the image and produce

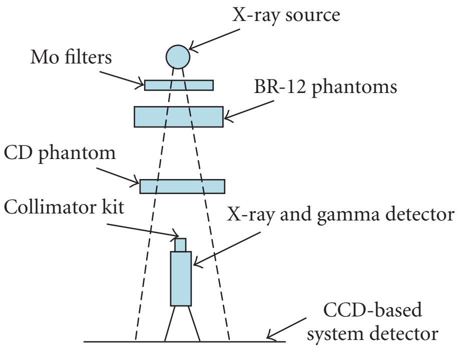

Figure 1: Schematic of spectra acquisition system.

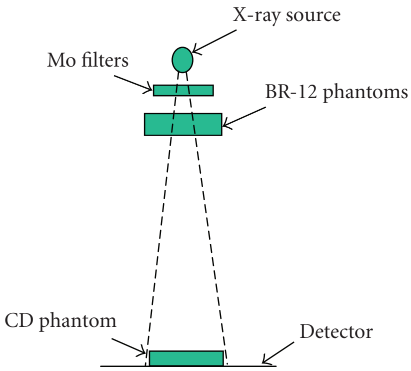

Figure 2: Schematic of contrast-detail phantom image acquisition system.

a high radiation dose. Conversely, the high-energy X-rays degrade the image quality by reducing the contrast. Spectra with a narrowband around the effective energy are preferred.

An *observer-based* contrast-detail (CD) curve is one of the important attributes of the X-ray digital imaging system. Its analysis is an effective, although subjective, method of evaluating image qualities [6, 7]. Combined objective and subjective studies can determine the quality of an X-ray imaging system. Based on the Rose model of human visual perception [8], an experimental technique to evaluate object detectability at the threshold of human visibility in medical images was developed [9, 10]. The detectability of low-contrast objects of various sizes is expressed graphically as a contrast-detail curve, which relates the threshold contrast necessary to perceive an object as a function of the object's size. When the threshold contrast is displayed versus the detail (object diameter), a typical contrast-detail curve begins at the upper left corner of the graph (i.e., high contrast, small detail) and declines asymptotically toward the right lower corner (i.e., low contrast, large detail) in the shape of a hyperbola [11].

A CCD-based digital imaging system was designed for small animal studies. The unique features of this system include an ultrasmall focal spot, hence providing a high spatial resolution [12]. This system has been used in studies of small animal vasculatures [13]. Preliminary observer-based analysis has shown that it is a useful tool for small animal studies [14]. In this study, both exit X-ray spectra and contrast-detail phantom images were acquired under various conditions of kVp, filter thickness, and phantom thickness. An *observer-based study* was conducted to evaluate the contrast-detail phantom images with different filtration conditions.

### 2. MATERIALS AND METHODS

A charge-coupled device (CCD)-based X-ray unit, designed for high-detail radiographic imaging (Faxitron X-Ray Corp, Wheeling, Ill) was used for this research. The X-ray tube of this CCD system has a focal spot of 20 $\mu$m and is designed to minimize the geometric blur introduced by magnification. The X-ray tube uses a tungsten target and a 0.254 mm beryllium window filtration to produce radiation with energies ranging from 10 kVp to 35 kVp. The tube current is fixed at 0.3 mA, with an exposure time ranging from 0.1 second to 999 seconds. The distance between the focal spot and the detector is 57.2 cm [15]. The detector module used in this system is composed of a Min-R medium mammography intensifying screen (Eastman Kodak, Rochester, NY) and two fiber optically coupled CCDs. The pixel size on the surface of the intensifying screen is 0.048 mm $\times$ 0.048 mm, with a $1024 \times 2048$ array and 12-bit digitization.

Table 1: The geometrical parameters of the tungsten collimators.

| Disc | Disc thickness (mm) | Hole diameter ($\mu$m) |
|---|---|---|
| 1 | 1 | 25 |
| 2 | 1 | 50 |
| 3 | 2 | 100 |
| 4 | 2 | 200 |
| 5 | 2 | 400 |
| 6 | 2 | 1000 |
| 7 | 2 | 2000 |

Observer-based measurements are conducted using a phantom specially designed for contrast-detail studies (Model 10001, MedOptics Corp., Tucson, Ariz). It is a $6 \times 6 \times 1.15$ cm Lucite slab with forty-nine holes arranged in a $7 \times 7$ matrix configuration. The holes have seven different diameters ranging from 0.18 to 4.82 mm. They also have different depths ranging from 0.06 mm to 0.73 mm. The contrast of the targets, which increases with the depth of the holes, changes along the rows, and the detail of the target, which increases with the diameter of the holes, changes along the columns [16]. Two breast tissue simulating phantoms, a 1 cm BR-12 phantom, and a 1.15 cm Lucite phantom (MedOptics Corp.) were used. The two phantoms have almost identical X-ray attenuation coefficient. These two phantoms were combined together to make a phantom with an equivalent thickness of 2.15 cm.

The X-ray spectra were obtained by using a spectrometer, which includes an XR-100T-CdTe X-ray and gamma detector system with a power supply and shaping amplifier (Amptek

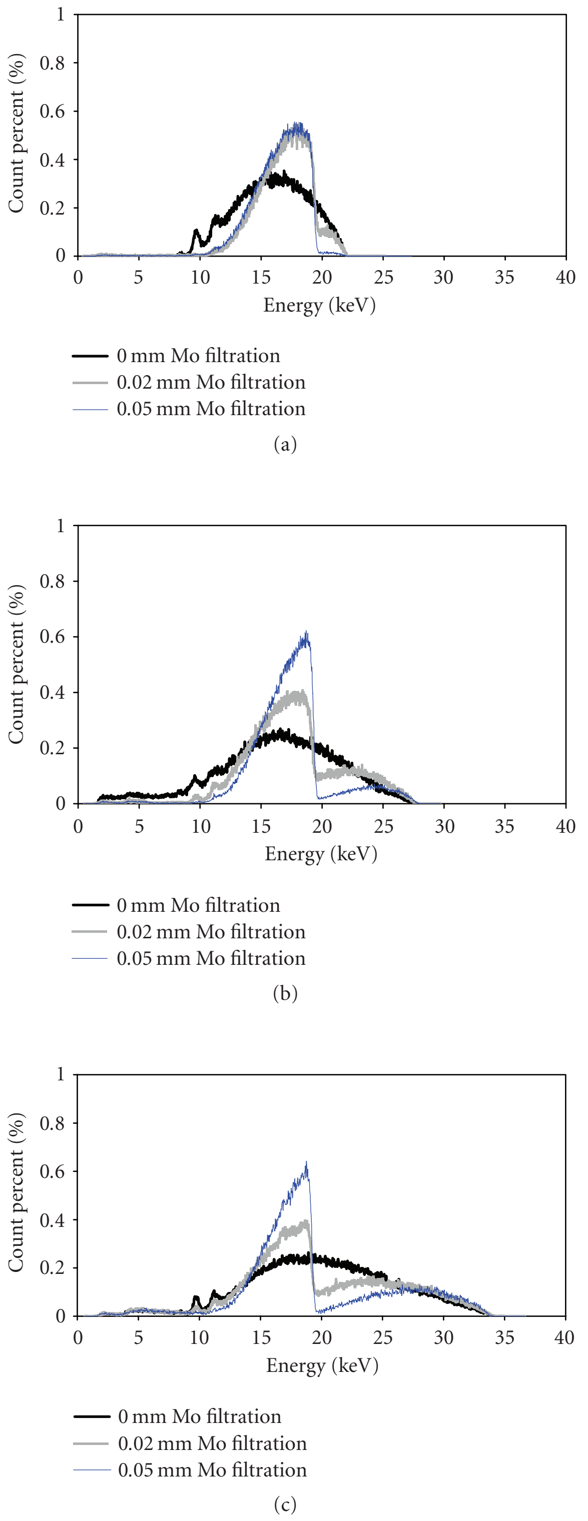

Figure 3: (a) X-ray spectra using the 1.15 cm phantom under the exposure of 22 kVp with a 0.0 cm (thick curve), 0.02 cm (gray curve), and 0.05 cm (thin curve) Mo filter. (b) X-ray spectra using the 1.15 cm phantom under the exposure of 28 kVp with a 0.0 cm (thick curve), 0.02 cm (gray curve), and 0.05 cm (thin curve) Mo filter. (c) X-ray spectra using the 1.15 cm phantom under the exposure of 35 kVp with a 0.0 cm (thick curve), 0.02 cm (gray curve), and 0.05 cm (thin curve) Mo filter.

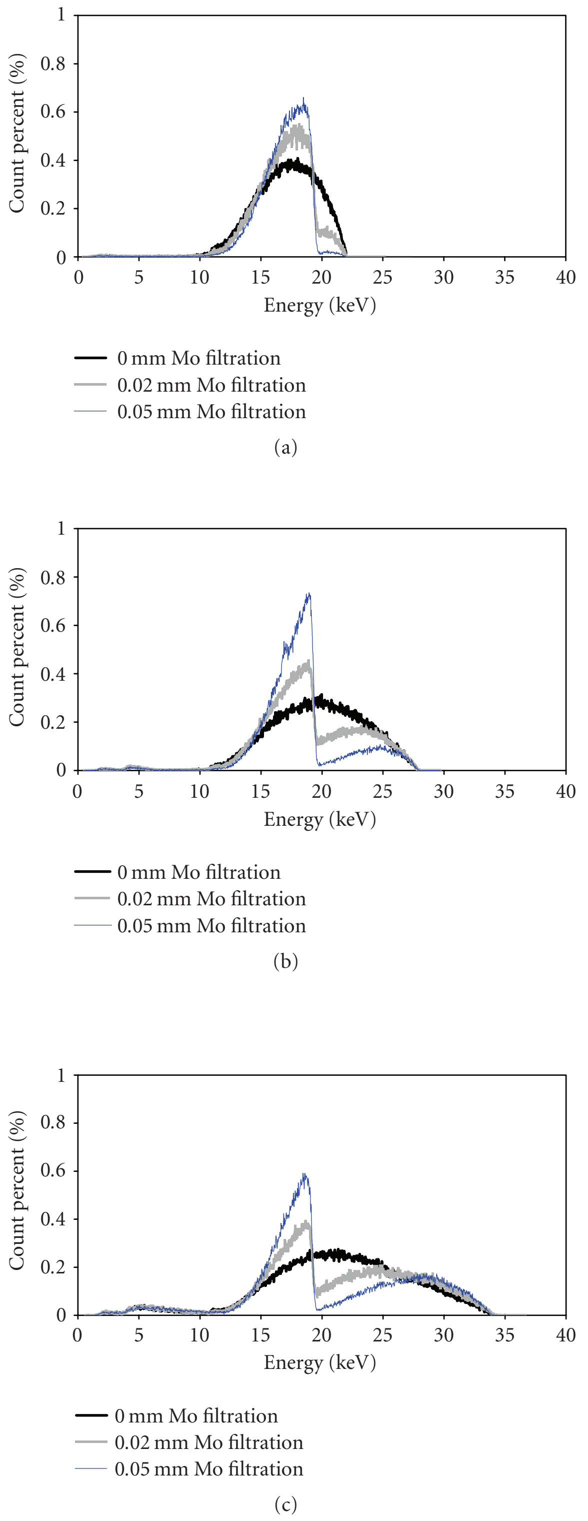

Figure 4: (a) X-ray spectra using the 2.15 cm phantom under the exposure of 22 kVp with a 0.0 cm (thick curve), 0.02 cm (gray curve), and 0.05 cm (thin curve) Mo filter. (b) X-ray spectra using the 2.15 cm phantom under the exposure of 28 kVp with a 0.0 cm (thick curve), 0.02 cm (gray curve), and 0.05 cm (thin curve) Mo filter. (c) X-ray spectra using the 2.15 cm phantom under the exposure of 35 kVp with a 0.0 cm (thick curve), 0.02 cm (gray curve), and 0.05 cm (thin curve) Mo filter.

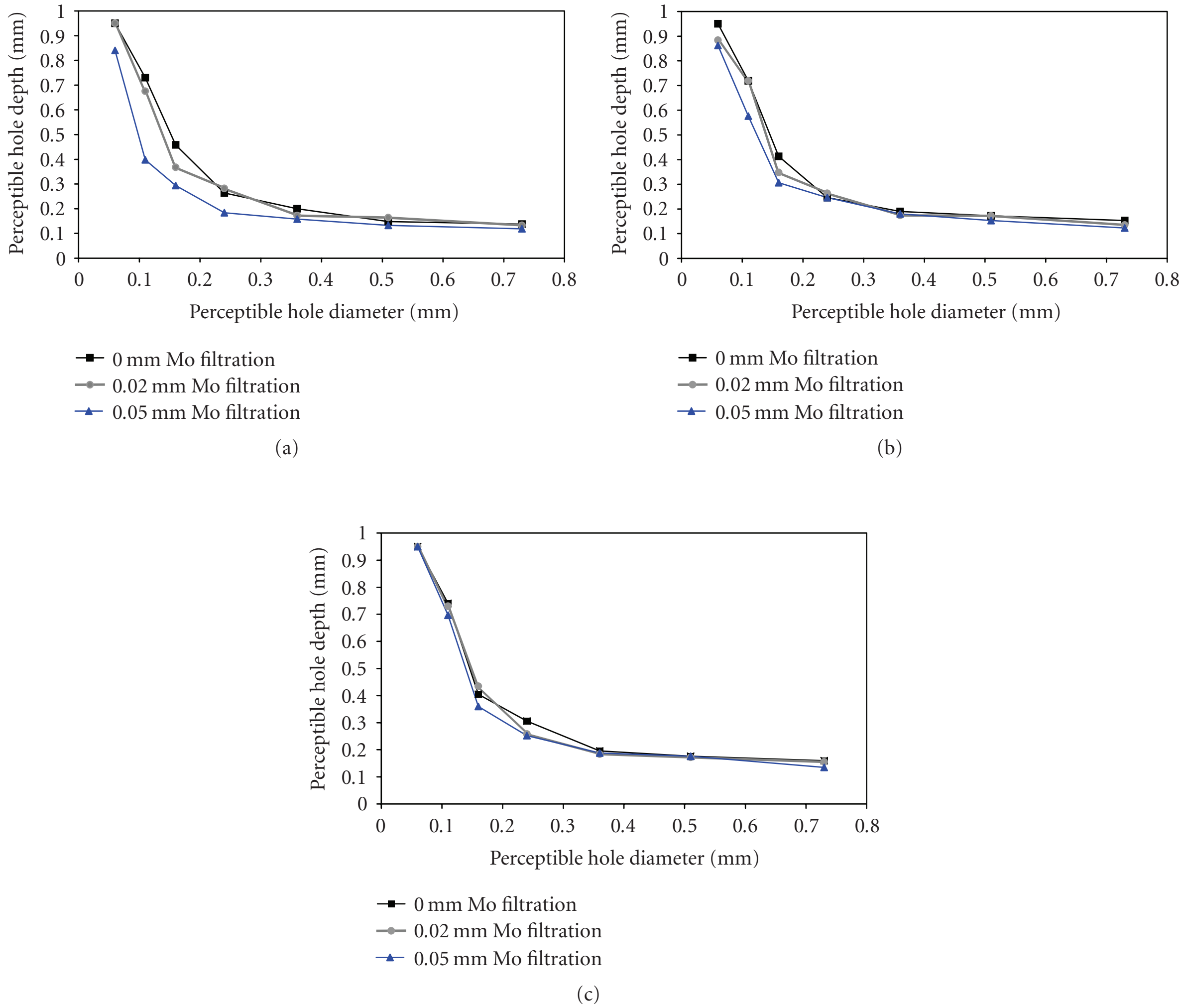

Figure 5: (a) Contrast-detail curves using the 1.15 cm phantom under the exposure of 22 kVp with a 0.0 cm (curve with squares), 0.02 cm (curve with circles), and 0.05 cm (curve with triangles) Mo filter. (b) Contrast-detail curves using the 1.15 cm phantom under the exposure of 28 kVp with a 0.0 cm (curve with squares), 0.02 cm (curve with circles), and 0.05 cm (curve with triangles) Mo filter. (c) Contrast-detail curves using the 1.15 cm phantom under the exposure of 35 kVp with a 0.0 cm (curve with squares), 0.02 cm (curve with circles) and 0.05 cm (curve with triangles) Mo filter.

Inc., Bedford, Mass). This unit is a high-performance X-ray and gamma ray detector equipped with a thermo-electric cooler [17].

The X-ray flux rate of the photons may exceed the capability of both the detector and the electronics that process the X-ray spectrum [18]. In order to reduce the count rate to an acceptable level, a collimator kit was used. The collimator kit includes a stainless steel collimator housing and 7 tungsten collimator discs which provide different size pinholes. The geometrical parameters of the discs are given in Table 1.

By selecting the appropriate tungsten collimators, the incoming X-ray flux can be reduced to a level at which the X-ray spectrum can be processed properly by the detector and electronics of the system [19]. In this study, appropriate collimators were used so that the absorbed radiation dose by the subject was the same under different X-ray settings.

Measurements of the spectra were acquired using phantoms of different thicknesses: 1.15 cm (MedOptics phantom) and 2.15 cm (combination of BR-12 phantom and MedOptics phantom). These measurements were conducted separately with combinations of different kVp values (22, 28, and 35) and different Mo filter thicknesses (0, 0.02, and 0.05 mm).

The Mo filters were held by a lead foil with a 10 cm diameter opening (the size of the X-ray window) and placed right beneath the X-ray source. The phantom was placed 46.2 cm above the detector plane. The detector of spectrometer was placed 28 cm below the phantom. The schematic for acquisition of X-ray spectra is shown in Figure 1.

For acquiring contrast-detail phantom images, the X-ray spectrometer was removed. The contrast-detail phantom was placed in contact with the image detector to minimize

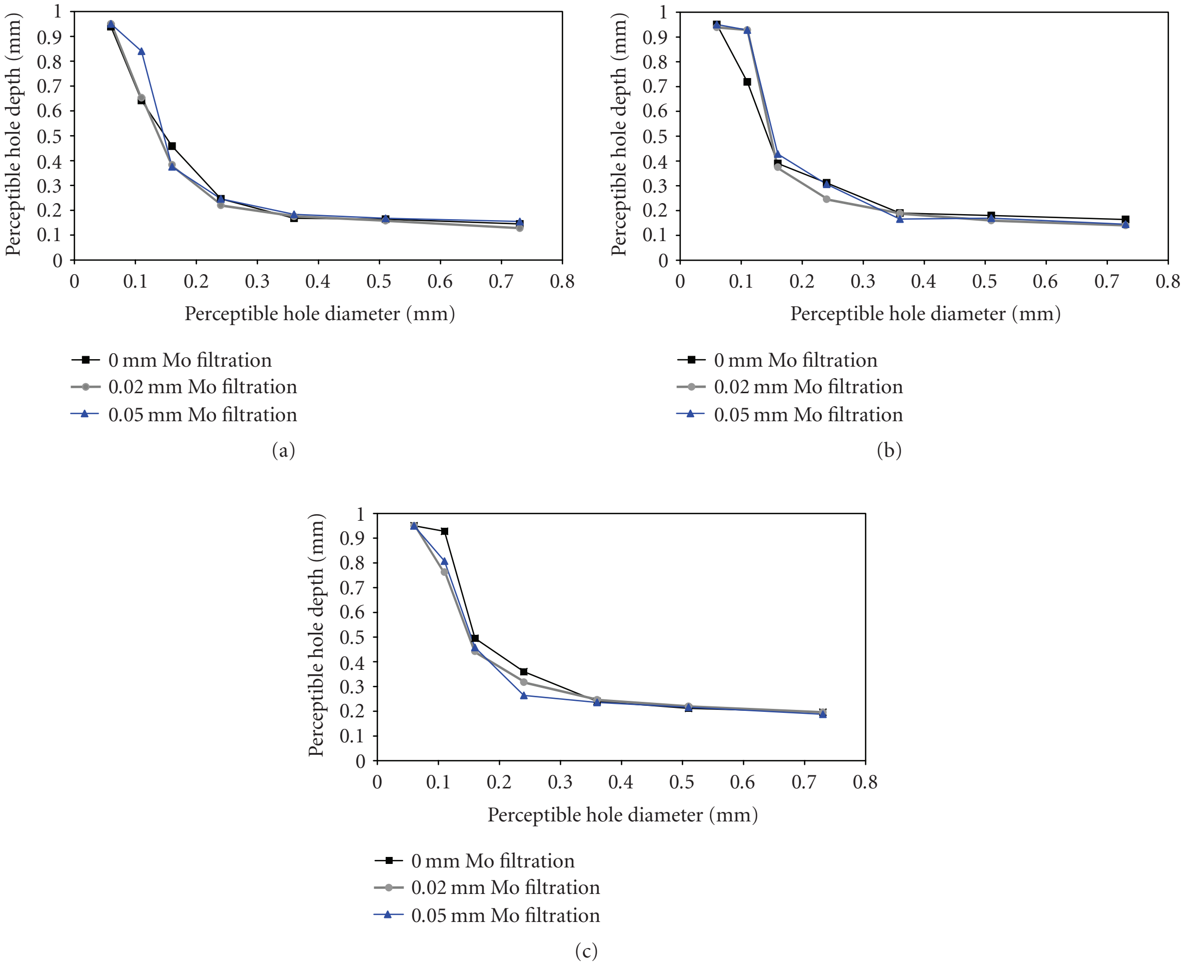

FIGURE 6: (a) Contrast-detail curves using the 2.15 cm phantom under the exposure of 22 kVp with a 0.0 cm (curve with squares), 0.02 cm (curve with circles), and 0.05 cm (curve with triangles) Mo filter. (b) Contrast-detail curves using the 2.15 cm phantom under the exposure of 28 kVp with a 0.0 cm (curve with squares), 0.02 cm (curve with circles), and 0.05 cm (curve with triangles) Mo filter. (c) Contrast-detail curves using the 2.15 cm phantom under the exposure of 35 kVp with a 0.0 cm (curve with squares), 0.02 cm (curve with circles), and 0.05 cm (curve with triangles) Mo filter.

magnification. By adjusting the exposure time, identical radiation exposure was obtained at the entrance of the image receptor in every condition. The exposure level was measured as 0.01 R by utilizing an X-ray exposure meter with a mammographic ion chamber (Rad-Check Plus, Victoreen, Inc., Cleveland, Ohio). The chamber was placed directly on top of the imaging detector. The exposure level of 0.01 R was selected because it resulted in an imaging signal that was in the middle of the dynamic range of the X-ray imaging module. Using the same combinations of the kVp and Mo filter thickness, a series of contrast-detail phantom X-ray images were obtained under the same exposure level for the observer-based study. The schematic of the filters and the phantom locations is shown in Figure 2. The images were presented on a CRT computer monitor, one meter away from observers in a dim room. The bright background of the monitor was masked with black paper. The images were processed with window leveling individually to achieve the best perceptible displayed quality. Once the window level was set, the observers were not allowed to make further adjustment of the image appearance on the display. Ten trained observers evaluated these images and recorded the minimum perceptible hole-depths.

### 3. RESULTS

The acquired spectrum was normalized to its total counts. Normalized count percentage = (count number of a given channel/ total count number) × 100%. Figures 3 and 4 show the X-ray spectra using the 1.15 cm and the 2.15 cm phantoms, respectively, under different energy levels (22, 28, and 35 kVp) and different Mo filtration thicknesses (0.0, 0.02, and 0.05 mm). The filtrations under all conditions provided significant reductions at both high- and low-energy regions.

The minimum perceptible hole-depths (directly related to threshold contrast) detected by the observers were

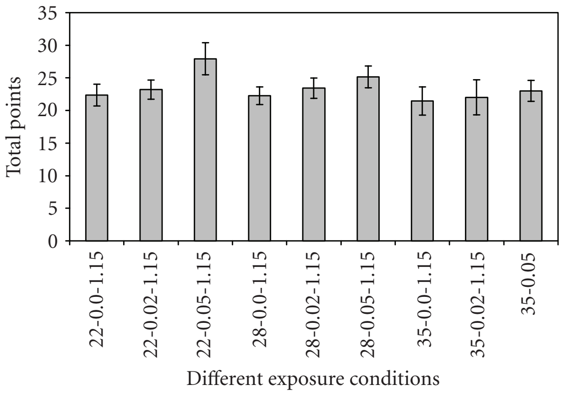

Figure 7: The average number of holes visible to all the observers under 9 different conditions with the 1.15 cm phantom. In each condition (the horizontal axis), the first number represents the X-ray energy (kVp) and the second number represents the thickness (in mm) of the Mo filter. It shows that the X-ray image taken using 22 kVp and 0.05 mm Mo filter has the highest total points (27.73). The error bars indicate the standard errors for the means of the points under each condition.

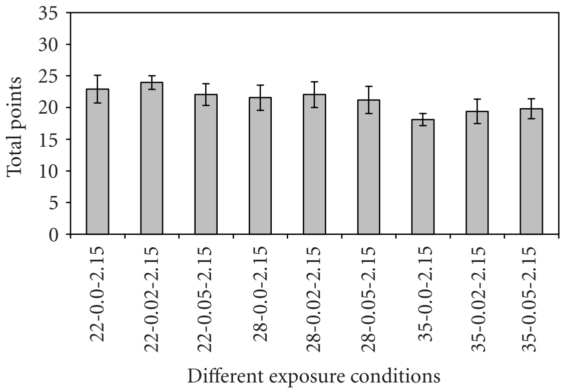

Figure 8: The average number of holes visible to all the observers under 9 different conditions with the 2.15 cm phantom. In each condition (the horizontal axis), the first number represents the X-ray energy (kVp) and the second number represents the thickness (in mm) of the Mo filter. It shows that the X-ray image taken using 22 kVp and 0.05 mm Mo filter has the highest total points (27.73). The error bars indicate the standard errors for the means of the points under each condition.

recorded as functions of the diameter of the holes (the details). Ten observers participated in the study and their results were averaged and plotted as contrast-detail curves, under different conditions. The contrast-detail curves using the 1.15 cm and the 2.15 cm phantoms are given in Figures 5 and 6, respectively. The observer-based contrast details were not significantly affected by the filtration, although at lower energy (22 kVp) the detectability of the subjects was noticeably improved by the filtration.

The total number of holes on the 49-hole MedOptics phantom visible to each observer was recorded. The results are shown in Figures 7 and 8 for the 1.15 cm and 2.15 cm phantoms, respectively. Using the results in these two figures, the quality of the X-ray images were compared. Using the 1.15 cm phantom, the observers detected most holes in the X-ray image acquired by 22 kVp and 0.05 mm Mo filter (average point: 27.73). Using the 2.15 cm phantom they detected most holes in the X-ray image acquired with 22 kVp and the 0.02 mm Mo filter (average point: 23.95).

### 4. DISCUSSION

In this study, Mo filters of different thicknesses were used in a digital X-ray imaging system for small animals research. Using this system, images of phantom simulating tissue of different thicknesses were acquired. The Mo filters effectively removed the low-energy portion of X-ray spectra, as shown in Figures 3 and 4. Since the low-energy X-ray photons only increase the patient radiation dose without much contribution to the contrast of the X-ray images, the reduction of such photons will reduce the radiation risks. Furthermore, the Mo filters provided a low cut-off around 10 keV, particularly when a 2.15 cm phantom was used (see Figure 4). At the higher-energy region, the Mo filters reduced the X-ray photons higher than 20 keV because of the K-edge of the Mo filters. Again, strong attenuation (2.15 cm phantom) significantly enhanced the cut-off above 20 keV (see Figure 4).

By comparing the results in Figure 3 (using the 1.15 cm phantom) and Figure 4 (using the 2.15 cm phantom), it is easy to notice that the thicker the filter and/or the phantom, the narrower the spectrum. The results in the spectrum study show that with appropriate filtration, the shape of the spectra become a narrowband. Furthermore, using the phantom of thickness in the range of tissue thickness of small animals, better image quality can be obtained with reduced radiation dose to the subjects. The results presented in this study provide a guide for selecting adequate filtration for subjects of different thicknesses.

The overall contrast details indicate that the filtration enhances detectability with the same absorbed radiation dose by the subject, particularly when the phantom is thin and the energy is low (see Figures 5(a) and 7). When the phantom is thick, the impact of filtration on detectability is not as significant, as shown in Figures 6 and 8.

Using the 49-hole phantom for contrast-detail analysis, the image acquired using the 1.15 cm phantom, with 22 kVp and the 0.05 mm Mo filter, has the highest number of detectable holes (see Figure 7). On the other hand, the image acquired using the 2.15 cm phantom, with 22 kVp and the 0.02 mm Mo filter, has the highest number of detectable holes (see Figure 8). Although the results we obtained are not conclusive in terms of the optimal exposure and filtration, the contrast-detail analysis and the X-ray spectra in this study indicate that with appropriate filtration and energy level, optimal imaging conditions can be obtained for subjects of different thicknesses, particularly in research involving small animals.

### ACKNOWLEDGMENTS

This work was supported in part by Grants from the National Institutes of Health (RO1 EB-002604, RO1 CA104773), by the College of Graduate Studies and Research, University of Central Oklahoma, and by a Grant from the National Institutes of Health (P20 RR016478 from the INBRE Program of the National Center for Research Resources). The authors would like to acknowledge the support of Charles and Jean Smith Chair endowment fund as well. The authors would also like to thank the help from observers Qiong Wang, Ben Steele, Yiyang Zhou, Yongsheng Ni, Xingwei Wang, and Da Zhang.

### REFERENCES

[1] M. Sandborg and G. A. Carlsson, "Influence of X-ray energy spectrum, contrasting detail and detector on the signal-to-noise ratio (SNR), and detective quantum efficiency (DQE) in projection radiography," *Physics in Medicine and Biology*, vol. 37, no. 6, pp. 1245–1263, 1992.

[2] L. J. Regano and R. A. Sutton, "Radiation dose reduction in diagnostic X-ray procedures," *Physics in Medicine and Biology*, vol. 37, no. 9, pp. 1773–1788, 1992.

[3] S. A. Beaman and S. C. Lillicrap, "Optimum X-ray spectra for mammography," *Physics in Medicine and Biology*, vol. 27, no. 10, pp. 1209–1220, 1982.

[4] R. Fahrig and M. J. Yaffe, "Optimization of spectral shape in digital mammography: Dependence on anode material, breast thickness, and lesion type," *Medical Physics*, vol. 21, no. 9, pp. 1473–1481, 1994.

[5] C. P. McDonagh, J. L. Leake, and S. A. Beaman, "Optimum X-ray spectra for mammography: choice of K-edge filters for tungsten anode tubes," *Physics in Medicine and Biology*, vol. 29, no. 3, pp. 249–252, 1984.

[6] X. J. Rong and C. C. Shaw, "Microcalcification detectability for four mammographic detectors: Flat-panel, CCD, CR, and screen/film," *Medical Physics*, vol. 29, no. 9, pp. 2052–2061, 2002.

[7] International Commission on Radiation Units Measurements, "Medical imaging—The assessment of image quality," ICRU Report 54, International Commission on Radiation Units and Measurements, Bethesda, Md, USA, 1996.

[8] A. Rose, "The sensitivity performance of the human eye on an absolute scale," *Journal of the Optical Society of America*, vol. 38, pp. 196–208, 1948.

[9] L. K. Wagner, G. Cohen, W.-H. Wong, and S. R. Amtey, "Dose efficiency and the effects of resolution and noise on detail perceptibility in radiographic magnification," *Medical Physics*, vol. 8, no. 1, pp. 24–32, 1981.

[10] G. Cohen, D. L. McDaniel, and L. K. Wagner, "Analysis of variations in contrast-detail experiments," *Medical Physics*, vol. 11, no. 4, pp. 469–473, 1984.

[11] H. Liu, L. L. Fajardo, J. R. Barrett, and R. A. Baxter, "Contrast-detail detectability analysis: Comparison of a digital spot mammography system and an analog screen-film mammography system," *Academic Radiology*, vol. 4, no. 3, pp. 197–203, 1997.

[12] F. Ouandji, E. Potter, W. R. Chen, Y. Li, D. Tang, and H. Liu, "Characterization of a CCD-based digital X-ray imaging system for small-animal studies: properties of spatial resolution," *Applied Optics*, vol. 41, no. 13, pp. 242–2427, 2002.

[13] C. Ye, Q. Luo, W. R. Chen, W. Liang, W. Luo, and A. Zhong, "Limitation in detecting internal blood vessels using digital X-ray imaging technique," *Journal of X-Ray Science and Technology*, vol. 12, no. 2, pp. 59–71, 2004.

[14] Q. Zhang, Y. Li, B. Steele, et al., "Comparison of a CMOS-based and a CCD-based digital X-ray imaging system: Observer studies," *Journal of Electronic Imaging*, vol. 14, no. 2, pp. 023002 (6 pages), 2005.

[15] MX-20 Specimen radiography system (CCD detector) technical manual.

[16] MedOptics contrast-detail phantom product description.

[17] Operating manual XR-100T-CdTe X-ray and Gamma ray detector system and PX2T power supply/shaping amplifier.

[18] Y. Kodera, H.-P. Chan, and K. Doi, "Effect of collimators on the measurement of diagnostic X-ray spectra," *Physics in Medicine and Biology*, vol. 28, no. 7, pp. 841–852, 1983.

[19] Amplex Collimator Kit Manual.

**Qirong Zhang** received his M.S. degree in electrical and computer engineering from the University of Oklahoma in 2004. She is an engineering scientist with comprehensive training and experience in medical imaging.

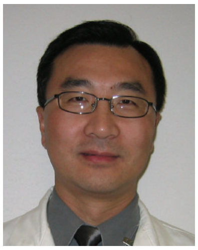

**John Rong** received his B.S. degree in nuclear physics from Peking University in 1984. He received his M.S. and Ph.D. degrees in health physics from University of Missouri-Columbia in 1992 and 1996, respectively. From 1997 to 1999, he was a postdoctoral fellow in radiation oncology physics at University of Michigan School of Medicine. In 1999 he joined the faculty of Diagnostic Radiology Department of the M.D. Anderson Cancer Center, where he specialized in digital x-ray imaging. He later completed his clinical residency in imaging physics at the M.D. Anderson Cancer Center in 2003. He then joined the University of Oklahoma Health Sciences Center as an Assistant Professor of radiology. He is certified in Diagnostic Radiologic Physics by American Board of Radiology and licensed in the State of Texas as a professional medical physicist. His clinical practice covers all aspects of medical imaging physics and his research interests focus on developing and evaluating new digital imaging techniques, optimizing imaging systems, and reducing patient radiation dose.

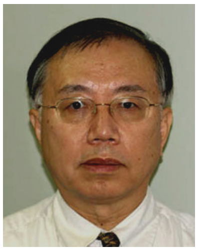

**Xizeng Wu** a medical physicist, received his Ph.D. in high-energy physics from the City University of New York in 1983. Currently he is a Professor of Radiology at University of Alabama at Birmingham, USA. His current interests are in X-ray phase-contrast imaging, and MRI with hyperpolarized spins.

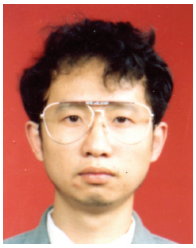

**Yuhua Li** received his PhD Degree in 1996, in optical information processing from Nankai University, China. From 1996 to 1999, he was a post doctoral fellow and an associate professor at Tianjin University, China, working on optical imaging processing and compact processor. From 1999 to 2001, he was a postdoctoral fellow at the University of South Florida and University of Texas at Austin, working on optical fiber high-temperature sensor, optical fiber communication and medical imaging processing. From 2001 to present, he is at the University of Oklahoma as a research associate, working on optical cardiac mapping and X-ray medical image system.

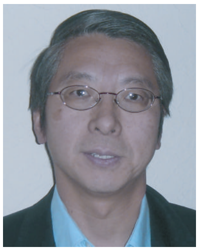

**Wei R. Chen** received his Ph.D. degree in physics from the University of Oregon, Eugene, Ore, in 1988. He is currently a Professor at the Department of Physics and Engineering and the Director of Biomedical Engineering program at the University of Central Oklahoma, and an Adjunct Associate Professor at the University of Oklahoma and Oklahoma State University. His main research interests include laser-tissue interactions, light transport in tissues, laser cancer treatment, and monitoring of cancer treatment using CCD-based digital X-ray imaging, magnetic resonance imaging, and other modalities. He is especially interested in laser photothermal and immunological effects in the treatment of metastatic tumors in preclinical and clinical studies. He has published more than 50 peer-reviewed research articles and has been awarded 8 US and international patents. His research has been sponsored by industry, private foundations, and by federal and state funding agencies.

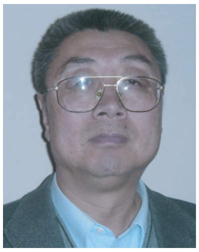

**Hong Liu** is currently the Charles and Jean Smith Chair in biomedical engineering, Professor of electrical and computer engineering, and Adjunct Professor of medicine at the University of Oklahoma. He is a Fellow of the American Institute of Medical and Biological Engineering (AIMBE), and a Fellow of the International Society for Optical Engineering (SPIE). He is also the Chief Editor of the Journal of X-ray Science and Technology. He is an active researcher in medical imaging. His current research projects include phase and phase contrast X-ray imaging, digital mammography, digital radiography, stereo fluoroscopy, and optical and fluorescent imaging devices. He has published more than 100 peer-reviewed scientific articles, numerous book chapters, and several patents. His research has been sponsored continually by major research grants from the National Institutes of Health (NIH) and other peer-reviewed funding agencies.

